# Development of a Hybrid Path Planning Algorithm and a Bio-Inspired Control for an Omni-Wheel Mobile Robot

**DOI:** 10.3390/s20154258

**Published:** 2020-07-30

**Authors:** Changwon Kim, Junho Suh, Je-Heon Han

**Affiliations:** 1 Daegu Research Center for Medical Devices and Rehabilitation, Korea Institute of Machinery and Materials, Daegu 42994, Korea; cwkim@kimm.re.kr; 2 School of Mechanical Engineering, Pusan National University, Busan 46241, Korea; junhosuh@pusan.ac.kr; 3 Mechanical Engineering, Korea Polytechnic University, 237. Sangidaehak-ro, Siheung-si, Gyeonggi-do 15073, Korea

**Keywords:** brain limbic system, fuzzy analytic hierarchy process, A*, omni-wheel mobile robot

## Abstract

This research presents a control structure for an omni-wheel mobile robot (OWMR). The control structure includes the path planning module and the motion control module. In order to secure the robustness and fast control performance required in the operating environment of OWMR, a bio-inspired control method, brain limbic system (BLS)-based control, was applied. Based on the derived OWMR kinematic model, a motion controller was designed. Additionally, an optimal path planning module is suggested by combining the advantages of A* algorithm and the fuzzy analytic hierarchy process (FAHP). In order to verify the performance of the proposed motion control strategy and path planning algorithm, numerical simulations were conducted. Through a point-to-point movement task, circular path tracking task, and randomly moving target tracking task, it was confirmed that the suggesting motion controller is superior to the existing controllers, such as PID. In addition, A*–FAHP was applied to the OWMR to verify the performance of the proposed path planning algorithm, and it was simulated based on the static warehouse environment, dynamic warehouse environment, and autonomous ballet parking scenarios. The simulation results demonstrated that the proposed algorithm generates the optimal path in a short time without collision with stop and moving obstacles.

## 1. Introduction

Mobile robots have become an essential element in production sites, medical sites, and various robot-based service environments. Various wheel-based driving methods have been developed to maximize work efficiency in environments wherein a mobile robot is applied [1]. In particular, due to the high maneuverability of the omnidirectional mobile robots, they are used in various fields/areas, such as warehouses, airports, shipbuilding companies, highly automated parking lots, etc. However, most of the work environment is not a stationary environment wherein an unchanging map-based work is possible, but it is a dynamic environment where the work environment is partially or significantly changed. Since the omnidirectional mobile robot is operated in a dynamic working environment, the functions to cover the following issues are essential for the mobile robot to respond to such environment. The first issue is to control the robot to the desired position in a dynamic environment wherein uncertainty exists. Therefore, mobile robots are required to have robustness against to the model uncertainty and sensor noise, fast responses to rapidly changing references, and error elimination performance to achieve high accuracy position control. The second aspect is to generate an efficient path to the assigned target without collision with the obstacles. Since the mobile robot is operated in a dynamic environment, an online path planning method is required that generates a path while adapting to a changing environment rather than generating an offline path that relies on a static map. In these respects, the motion control and path planning are two core issues to study in the omnidirectional robot field.

In order to control an omnidirectional robot, reference [2] applied a PID control scheme for the omni-wheel mobile robot (OWMR) position and velocity control. Reference [3] also proposed a PID controller taking into account the dynamics of actuators and the actuator saturation while designing the controller. Reference [4] suggested the trajectory linearization control method based on both the kinematics and dynamics of an omnidirectional mobile robot. Reference [5] designed a steerable omnidirectional wheel-based mobile robot and controlled the motion via PI control. By introducing velocity and acceleration filters, reference [6] archived a precise control performance under the robot’s position error. Reference [7] proposed a model-predictive controller with input and output state constraints included. To improve the obstacle avoidance performance of a remotely controlled OWMR, reference [8] suggested the EMG (electromyography) and artificial potential field hybrid controller.

In the area of path planning, a lot of research have been done on multi-objective based path planning because it is necessary to consider several aspects simultaneously, such as travel distance, collision safety, and agility of the path, rather than simply generating a path considering only a single factor. Castillo et al. [9] applied the multi-objective genetic algorithm while defining travel distance and travel difficulty of the path as the objectives. Masehian and Sedighizadeh [10] considered shortness and smoothness as the objectives by combining particle swarm optimization and a probability road map. Ahmed and Deb [11] modified the non-dominated sorting genetic algorithm II while taking into account travel distance, safety, and path smoothness simultaneously. More recently, references [12,13] suggested multi-objective considered path planning strategies. Some of the researchers utilized the multi-objective decision-making (MODM) method in path planning. Kim and Langari [14] utilized the analytic hierarchy process (AHP) to plan an optimal path of a mobile robot. Kouzehgar et al. [15] introduced a simple additive weighting (SAW)-based path planning strategy while considering area coverage and energy consumption as the considering objectives for a cleaning robot. Reference [16] suggested full consistency method (FUCOM) theory to plan a path considering four objectives: the convenience of the terrain configuration for robots motion, the risk of communication loss with the cloud, the risk to meet the human-robot interactions, and the robot safety related to conditions dependent on each specific mission.

Most OWMR operations-related studies cover only one aspect, either motion control or route planning. On the other hand, references [17,18] considered two issues at the same time. Additionally reference [19] proposed a surface EMG signal-based shared controller. The motion control is conducted by a human’s forearm EMG signal and the obstacle avoidance is accomplished by no-target bug algorithm. However, in order to successfully operate an OWMR in a dynamic working environment, two aspects must be considered simultaneously. Therefore, in this study, a motion control function was developed through brain limbic system-based control, and the path planning algorithm is proposed through A* and the FAHP hybrid path generation method. The contribution of this research is to propose an overall control structure for OWMR that enables optimal path generation and motion control in a short-time under a dynamic working environment. The control structure includes the mobile platform motion controller as the lower-level controller and an online path planner as the higher-level controller. Each control level is developed to achieve dynamic working environment-applicable performance. A bio-inspired control strategy, brain limbic system-based control, showing control performance suitable for a dynamic environment, is suggested as a motion controller. In addition, a novel path planning algorithm is proposed. A suitable path planning algorithm should have characteristics of optimal path generation, short path generation time, and responsiveness to changing environments. A* can guarantee optimal path generation, but it takes a long time to generate a path. FAHP has characteristics suitable for a dynamic working environment, but it is not guaranteed to generate an optimal path. In this study, by combining these two offline and online route planning algorithms, a hybrid path planning algorithm that is suitable for a dynamic work environment is proposed. Through the simulation under various OWMR operation scenarios, the dynamic environmental suitability of the proposed control structure is demonstrated.

The organization of this paper is as follows. Section 2 provides the description of the problem. In Section 3, a bio-inspired controller for an OWMR is introduced. The hybrid path planning algorithm is proposed in Section 4. The performances of the suggested motion control strategy and path planning algorithm are demonstrated by numerical simulations in Section 5. Finally, the paper ends with some concluding remarks in Section 6.

## 2. Problem Description

### 2.1. Problem Description

**Figure 1:**
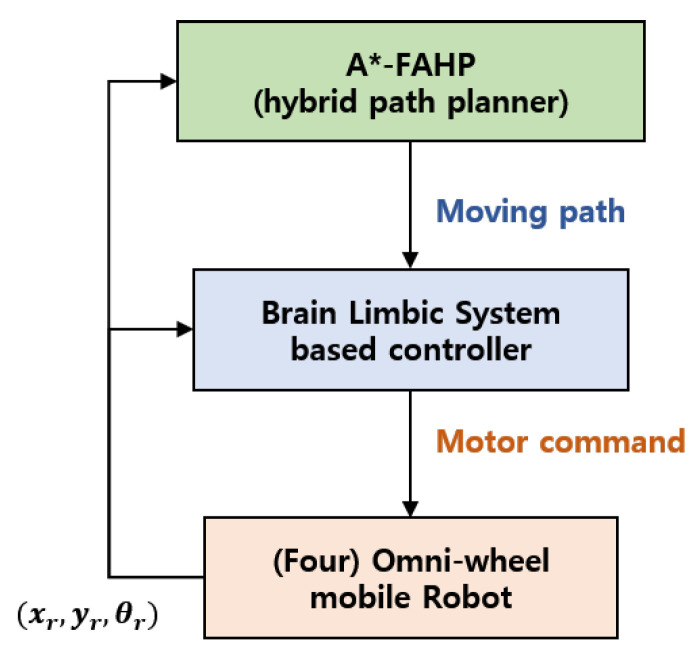
The schematic diagram and coordinate system of the parking robot.

The purpose of this research was to develop a control structure for an OWMR to be operated under a dynamic environment that plans the path and moves the robot to the desired position. Therefore, the controller consists of the higher-level controller and lower-level controller, as shown in Figure 1.

The higher-level controller generates a collision-free path to the target, and the lower-level controller drives the robot to the desired position. In order to implement the higher-level controller, the A*–FAHP hybrid path planning algorithm is suggested. The combination of offline (A*) and online (FAHP) path planning achieves a rapid, optimal path generation performance that copes with a dynamic environment. Additionally, the lower lever controller was developed based on the brain limbic system-based control. In order to design the motion controller, the kinematics of the four-wheeled OWMR were derived. The performance of the proposed control structure was verified through numerical simulations. The kinematics of OWMR were derived to reflect the characteristics of OWMR driving, and the performance of the motion controller was demonstrated in scenarios of point-to-point movement, circular path tracking, and random path movement target tracking. The performance of the path planning algorithm was tested in static warehouse environments, dynamic warehouse environments, and autonomous valet parking scenarios.

### 2.2. The Design of an OWMR Motion Controller

A bio-inspired control scheme, brain limbic system-based control, is proposed as an OWMR motion controller. In the mammalian brain, especially the limbic system, motion control is made based on a mechanism to learn appropriate behavior for a particular emotion. Moren and Balkenius [20] introduced a mathematical model that describes the physical phenomenon of the emotional processing and learning in the mammal brain. Since then, several researchers have applied neural emotion learning algorithms to feedback control problems. Lucas et al. [21] applied the algorithm in control system design. The brain limbic system (BLS) has been applied to various fields [22,23,24,25,26,27,28,29] due to its advantages such as the fast response, error elimination characteristics, and robustness against disturbances. Since the omni-directional robot is also deployed and operated in dynamic environments, the application of the BLS controller having the above-described characteristics is suitable.

### 2.3. A Hybrid Path Planning Algorithm

In this study, a multi-objective decision-making tool, fuzzy-based AHP, is utilized. AHP is a multi-purpose decision-making technique developed by Saaty [30] and it is applied in various decision-making fields, such as offshore manufacturing plant location [31], selecting a nuclear reactor type [32], mobile robot control [33], Web service selection [34], and traffic assessment [35] because of the following advantages. AHP is able to set the relative importance of considering objectives; able to model a given problem simply and flexibly; able to measure the consistency in decision-making; able to improve computational efficiency due to the simple calculation process [36]; and suitable for complex problems [37]. In addition to AHP, various MODM methods such as SAW, TOPSIS, and FUCOM have been developed. However, this study applied AHP, which is the most widely used and is easy to apply to real systems. In order to maximize the utilization of the mobile robot, it is essential to generate a path for the dynamic environment. FAHP has the ability to respond to dynamic aspects, such as unknown obstacles and moving targets, as shown in [36,38]. FAHP originated form analytic hierarchy process (AHP), which is one of the most famous multi-purpose decision-making tools and is used in various fields. In FAHP, the problem of ambiguity and uncertainty of AHP is improved through fuzzy theory. Chan and Kumar [39] showed that FAHP not only handles the uncertainty imposed by the decision maker during the decision-making process, but it also provides the robustness and flexibility. Laarhoven and Pedrycz [40] developed FAHP to quantitatively include the subjectivity of the decision maker in the decision-making process throughout a combination of triangular membership function and triangular fuzzy number. Then, Chang [41] proposed the extent analysis method, thereby replacing Saaty’s nine-point pairwise scale to triangular fuzzy numbers. References [36,38] applied FAHP on mobile robot path planning considering multi-objectives such as the travel distance to the target, collision safety with obstacles, and the rotation of the robot to face the target. However, due to the nature of the algorithm, FAHP, which is an online path planning algorithm, cannot guarantee optimal path generation. Therefore, a well-known optimal path planning algorithm A* is combined with FAHP to generate the optimal path.

## 3. A Bio-Inspired Controller Design for an Omni-Wheel Mobile Robot

In this section, a bio-inspired control method, BLS-based control, is utilized to control an OWMR. This section includes the kinematics of the OWMR, the structure of BLS, and the BLS controller’s design.

### 3.1. Kinematics of OWMR

**Figure 2:**
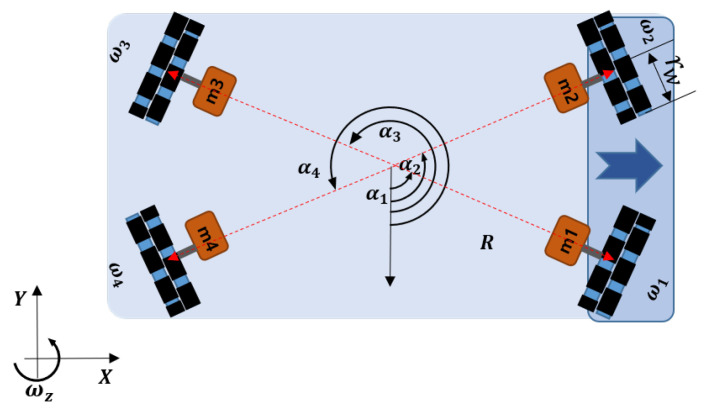
The schematic diagram and coordinate system of the parking robot.

Perpendicular driving and rotating in place are essential functions to maximize spacial efficiency. For the implementation of these functions, an OWMR platform is essential due to its maneuverability. In order to move the OWMR omni-directionally, all wheels are controlled independently. Figure 2 shows the schematic diagram of a four-omni-wheel-equipped mobile robot. It is assumed that the robot has four motors, $m_{1}$, $m_{2}$, $m_{3}$, and $m_{4}$, and wheels with a wheel radius of $r_{w}$. Each wheel is assembled with the same distance *R* from the center of the robot and with different angles $\alpha_{1}$, $\alpha_{2}$, $\alpha_{3}$, and $\alpha_{4}$, respectively.

(1) $$V_{\omega }=\frac{1}{r}\left[\begin{array}{ccc} -\sin\alpha_{1} & \cos\alpha_{1} & R\\ -\sin\alpha_{2} & \cos\alpha_{2} & R\\ -\sin\alpha_{3} & \cos\alpha_{3} & R\\ -\sin\alpha_{4} & \cos\alpha_{4} & R \end{array}\right]v=JV,$$

where *J* represents the Jacobian matrix of the OWMR and the attachment angles of each motor are $\alpha_{1},\alpha_{2},\alpha_{3},\alpha_{4}$. The robot position is given as (x,y,θ); therefore, the forward kinematics of the OWMR are required and it can be derived as the inverse form of Equation (1). However, due to *J* is not a symmetric matrix, a pseudo-inverse matrix of *J* is used as follows [42]:

(2) $$J^{+}={\left(J^{T}J\right)}^{-1}J^{T}.$$

By applying Equation (2), the forward kinematics of an OWMR are derived as follows:

(3) $$V=J^{+}V_{\omega }=\frac{r}{2}\left[\begin{array}{cccc} -\sin\alpha_{1} & -\sin\alpha_{2} & -\sin\alpha_{3} & -\sin\alpha_{4}\\ \cos\alpha_{1} & \cos\alpha_{2} & \cos\alpha_{3} & \cos\alpha_{4}\\ \frac{1}{2R} & \frac{1}{2R} & \frac{1}{2R} & \frac{1}{2R} \end{array}\right]\left[\begin{array}{c} \omega_{1}\\ \omega_{2}\\ \omega_{3}\\ \omega_{4} \end{array}\right],$$

utilizing the OWMR model, Equation (3), the BLS controller generates angular speed for each wheel to track OWMR’s desired position.

Using the coordinate system and by defining angular velocity of each wheel, $V_{\omega }={\left[\begin{array}{cccc} \omega_{1} & \omega_{2} & \omega_{3} & \omega_{4} \end{array}\right]}^{T}$ and the velocity vector of the robot $V={\left[\begin{array}{ccc} v_{x} & v_{y} & \omega_{z} \end{array}\right]}^{T}$, the kinematics of the OWMR [43,44] is given:

### 3.2. BLS-Based OWMR Motion Controller Design

**Figure 3:**
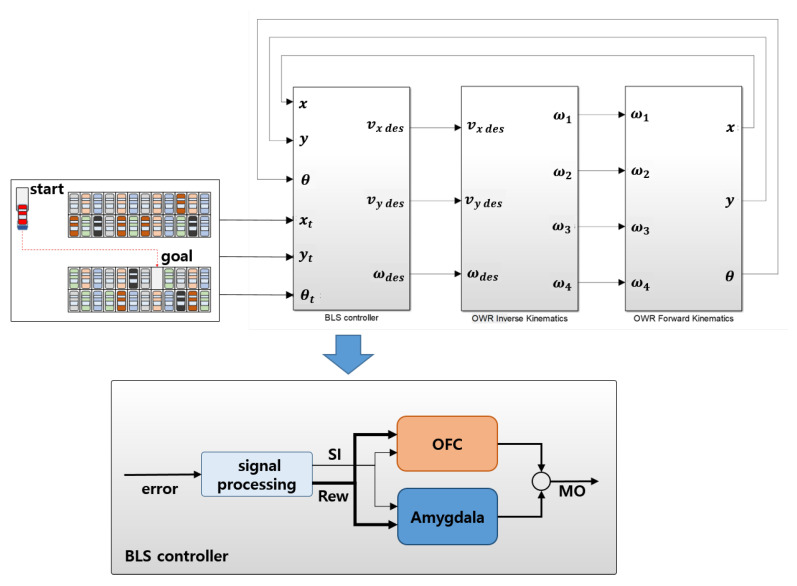
The lower-level control structure of the parking robot.

In this section, the design of the BLS-based control algorithm as a motion controller is described. It is a bio-inspired controller that mimics the emotion-based behavioral control that takes place in the mammalian brain. Because the possible applications of an OWMR are various, such as production, logistics, and even robot-based service, the motion controller is required to have robustness against disturbances, elimination of position error, and fast responses. From the previous researches, the main advantages of the BLS-based control include robustness to parameter uncertainty [26], error elimination [45], and fast response [46]. The BLS learns appropriate reactions to a given emotion. When stimuli are given from the outside, appropriate responses are learned based on the emotions generated by the stimuli. As the main components of the BLS, the amygdala, orbito-frontal cortex (OFC), sensory cortex, and thalamus are involved in the emotional processing and learning. The amygdala learns the appropriate relations between neutral and emotionally-charged stimuli while the OFC tries to inhibit inappropriate links as the task is accomplished. The thalamus is a communication path between the cortical and the other parts of the loop. The sensory cortex reprocesses the sensed input to produce SI from the definition of raw sensory input. Moren and Balkenius [20] developed a computational model of the brain limbic system (BLS) process. Equations (4) through (6) describe a simplified model of the process [27]:

(4) $$MO=\sum_{i}\left(A_{i}-OFC_{i}\right)$$

(5) $$A_{i}=G_{A_{i}}\cdot SI_{i}$$

(6) $$OFC_{i}=G_{OFC_{i}}\cdot SI_{i},$$

where MO is the output of the BLS controller and the subscript *i* represents the *i*-th sensing stream. $G_{A_{i}}$, $G_{OFC_{i}}$, and $SI_{i}$ represent the gains of amygdala, OFC, and sensory input, respectively. This learning strategy is inspired by biology, where the amygdaloid gains, $G_{A_{i}}$, are learned proportionally to the difference between the reward (where it could be the punishment, Rew) and the output signal of the amygdala nodes:

(7) $$\Delta G_{A_{i}}=\alpha \cdot SI_{i}\cdot max\left(0,Rew-\sum_{i}A_{i}\right),$$

where $\Delta G_{A_{i}}$ is the update rule of $G_{A_{i}}$ and α is the learning rate, which can be set to between 0 (no learning) and 1 (instant adaptation). In practice, it is usually set at a fairly low value. The learning of the amygdala nodes is a function of the learning rate α, the difference between the reward and the amygdala output, and the strength of the SI. The stronger the stimulus and the larger the difference between Rew and amygdala output, the faster the learning occurs. By taking the maximum value between 0 and the difference between Rew and $\sum_{i}A_{i}$, once learned, it is permanent and gives the system the ability to retain emotional connections. However, the OFC learning is not constrained to be monotonic:

(8) $$\Delta G_{OFC_{i}}=\beta \cdot SI_{i}\left(\sum_{i}A_{i}-\sum_{i}OFC_{i}-Rew\right),$$

where $\Delta G_{OFC_{i}}$ is the update rule of $G_{OFC_{i}}$ and β is the learning rate. It is note that the BLS is an online learning algorithm and influenced by the learning rate of amygdala and OFC. Therefore, the learning rate of the amygdala is set to be lower than that of OFC to avoid impulsive decisions on the part of the controller. It generates larger OFC output and inhibits the inappropriate response of the controller faster. The OFC tracks the mismatch between the system’s predictions and the actual received reward, and learns to inhibit the system’s output in proportion to the mismatch. By the observation of Equations (7) and (8), it is noted that the amygdaloid gains are not affected where no reward occurs; however, the OFC gain rapidly increases and inhibits the output. According to the update rules of amygdala and OFC, the output signals of the amygdala, OFC, and MO are obtained following Equations (4)–(6). As shown in Equations (4) and (5), SI and Rew perform significant roles in BLS. Because the framework of BLS-based controller is firmly defined, the main task of utilizing BLS control is designing the SI and reward function with respect to the purpose of the control. Figure 3 shows a autonomous parking lot wherein the OWMR is controlled via BLS control structure. It is assumed that the target position is assigned to the OWMR type parking robot; the BLS controller calculates the input vector *V* according to the position errors.

**Figure 4:**
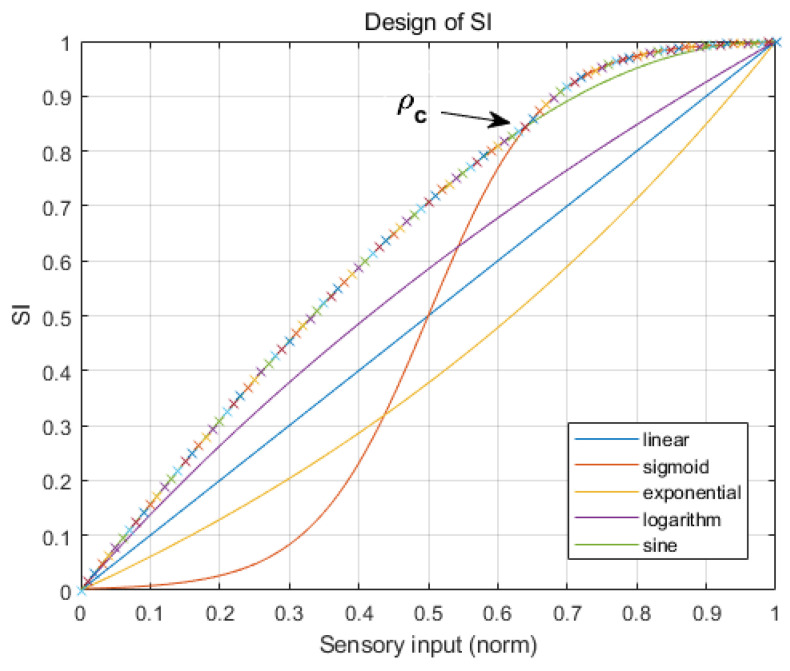
Functions for design SI.

The velocity vector is composed of translation speed ($v_{x}$, and $v_{y}$) and angular speed ($\omega_{z}$) of the OWMR. To design a BLS-based motion controller, the sensory inputs ($SI=\{SI_{v},SI_{\omega }\}$) and the rewards ($Rew=\{Rew_{v},Rew_{\omega }\}$) need to be defined. The distance error and angular error are modified to define the sensory input, SI. In addition, the normalized errors are reprocessed by several functions, as shown in Figure 4. In order to maximize the response performance of the control system, the highest value function is selected as the SI design function; i.e., if a normalized value is greater than $\rho_{c}$, the sigmoid function is used; otherwise, the sine function is selected as the SI design function. As a result, a combination of functions marked with an “x” was selected to generate the SI in Figure 4.

According to the selected SI design function, the $SI_{v}$ is defined as follows:

(9) $$SI_{v}=\left\{\begin{array}{cc} sin(\frac{\pi }{2}\rho_{v}), & \mathrm{if}\;(0\leq \rho_{v}\leq \rho_{v_{c}})\\ \frac{1}{1+e^{-12(\rho_{v}-0.5)}}, & \mathrm{if}\;(\rho_{v_{c}}<\rho_{v}\leq 1) \end{array}\right.,$$

where $\rho_{v}$ and $\rho_{v_{c}}$ represent the normalized distance error and intersecting between the sine function and sigmoid function, respectively. In the same way, the $SI_{\omega }$ is defined as follows:

(10) $$SI_{\omega }=\left\{\begin{array}{cc} sin(\frac{\pi }{2}\rho_{\omega }), & \mathrm{if}\;(0\leq \rho_{\omega }\leq \rho_{\omega_{c}})\\ \frac{1}{1+e^{-12(\rho_{\omega }-0.5)}}, & \mathrm{if}\;(\rho_{\omega_{c}}<\rho_{\omega }\leq 1) \end{array}\right.,$$

where $\rho_{\omega }$ and $\rho_{\omega_{c}}$ represent the normalized angular error and intersection between the sine function and sigmoid function, respectively. Then the $Rew_{v}$ and the $Rew_{\omega }$ are defined as follows:

(11) $$Rew_{v}=\left\{\begin{array}{cc} SI_{v}+\frac{1}{SI_{v}\cdot \int MO_{v}dt}, & \mathrm{if}\;(SI_{v}>0)\\ c_{v}, & \mathrm{if}\;(SI_{v}=0) \end{array}\right.,$$

where $MO_{v}$ and $c_{v}$ represent the translation speed control output and an arbitrary, non-zero, positive constant, respectively.

(12) $$Rew_{\omega }=\left\{\begin{array}{cc} SI_{\omega }+\frac{1}{SI_{\omega }\cdot \int MO_{\omega }dt}, & \mathrm{if}\;(SI_{\omega }>0)\\ c_{\omega }, & \mathrm{if}\;(SI_{\omega }=0) \end{array}\right.,$$

where $MO_{\omega }$ and $c_{\omega }$ represent angular speed control output and an arbitrary, non-zero, positive constant, respectively.

## 4. The A* and Fuzzy Analytic Hierarchy Process-Based Hybrid Path Planning Algorithm

In this section, a hybrid path planning algorithm is proposed. Recently, reference [36,38] developed an FAHP-based path planning algorithm while applying a single robot and multi-robot navigation scenarios, respectively. From these studies, FAHP considered multi-objectives such as distance to the target, safety, and rotation to the target simultaneously. The simulation results demonstrated the superiority of the FAHP. However, as an online path planning algorithm, FAHP generates a driving path route incrementally in response to a given environment without the map information of the entire workspace. When the goal is given, the FAHP selects the best candidate between all available alternatives considering the three objectives. In other words, FAHP is locally optimal at best by taking partial information about the working environment. Though the strength of FAHP is to cope with dynamic environments such as unexpected obstacles and moving objects well, due to the limitation of offline path planning algorithm, the optimality of the path cannot be guaranteed. A comparison between online and offline path planning algorithms [47] is well shown in Table 1. In this study, to secure the optimality of the FAHP-based path planning method, A*, an offline path planning method, was combined with FAHP. By combining the advantages of the online and offline methods, an optimal path planning method capable of responding to a dynamic environment was created.

In previous studies, FAHP was applied to the mobile robot path planning problem. Additionally, the A* algorithm is a well-known algorithm. Therefore, in this paper, two algorithms are briefly introduced, and more detailed descriptions of FAHP and A * are replaced by a reference [36,38,48].

### 4.1. FAHP Based Path Planning

FAHP is a multi-objective decision-making tool. The original version, the AHP (analytic hierarchy process), was developed by Satty [30], and to improve the AHP, reference [41] suggested the extent analysis method on fuzzy AHP using triangular fuzzy numbers.

**Figure 5:**
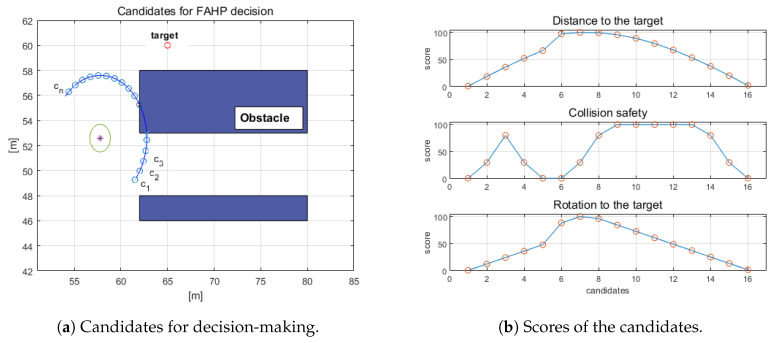
Definitions of candidates and the scores for FAHP-based decision-making.

Figure 5 displays a FAHP-based decision-making situation for path planning. It assumed a mobile robot equipped with a LiDAR sensor with a sensing radius of *R* m. Additionally, it was moving toward a target. The half-circle represents the LiDAR sensing boundary and the small circles are candidates for next movement of the robot. All the candidates were evaluated with objectives such as travel distance to the target, collision safety, and the rotation to the target. As shown in Figure 5b, candidates were given different scores according to the objectives considered. By means of FAHP, the best candidate was selected as the solution for the robot to move. The implementation of the FAHP follows the procedure below (the following were reduced from [38]).

- Step 1: defining relative importance among objectives.

(13) $$\mathrm{RM}=\left[\begin{array}{ccc} O_{1}/O_{1} & O_{1}/O_{2} & O_{1}/O_{3}\\ O_{2}/O_{1} & O_{2}/O_{2} & O_{2}/O_{3}\\ O_{3}/O_{1} & O_{3}/O_{2} & O_{3}/O_{3} \end{array}\right],$$

where $O_{n}$ means the *n*-th objectives. *RM* defines relative importance between objectives; i.e., $O_{1}/O_{2}=a$ indicates that $O_{1}$ is “*a*” times important than $O_{2}$, and $O_{2}/O_{3}=b$ shows that $O_{2}$ is “*b*” times important than $O_{3}$.

- Step 2: Consistency check of the relative important matrix.

However, the *RM* could be inappropriate because it is defined by the user’s preference. Therefore the consistency of *RM* should be examined by the AHP-based consistency method. It is also possible to estimate the departure from consistency by the consistency index (*CI*):

(14) $$\mathrm{CI}=\frac{\lambda_{max}-n}{n-1}.$$

To obtain the consistency ratio (*CR*), the *CI* is divided by the random consistency (*RC*) index as follows:

(15) $$\mathrm{CR}=\frac{\mathrm{CI}}{\mathrm{RC}}.$$

Saaty [30] limits the appropriate measure, as denoted by the *CR*, should not exceed 0.1. Only the case that meets the condition can be accepted.

- Step 3: Fuzzification of the relative important matrix.

Using the triangular fuzzy number, that fuzzified, relative importance matrix of Equation (13) is defined as follows.

(16) $$\mathrm{FRM}=\left[\begin{array}{ccccccccc} {}[1 & 1 & 1] & [a-d & a & a+d] & [b-d & b & b+d]\\ {}[\frac{1}{a+d} & \frac{1}{a} & \frac{1}{a-d}] & [1 & 1 & 1] & [c-d & c & c+d]\\ {}[\frac{1}{b+d} & \frac{1}{b} & \frac{1}{b-d}] & [\frac{1}{c+d} & \frac{1}{c} & \frac{1}{c-d}] & [1 & 1 & 1] \end{array}\right],$$

where FRM is fuzzified relative importance matrix.

- Step 4: Calculation of fuzzy synthetic extent.

The fuzzy synthetic extent [41] of FRM is calculated as follows.

(17) $$S_{i}=\sum_{j=1}^{m}FRM_{g_{i}}^{j}⊙{\left[\sum_{i=1}^{n}\sum_{j=1}^{m}FRM_{g_{i}}^{j}\right]}^{-1},$$

where $S_{i}$ is the *i*-th synthetic extent and all the $FRM_{g_{i}}^{j}$ are triangular fuzzy numbers.

- Step 5: Weight vector of FRM calculation.

After the fuzzy synthetic extent is obtained, the weight vector of defined objectives is derived. By the comparison principle of the fuzzy numbers [49], the normalized weight vector is given:

(18) $$W_{obj}={\left(d\left(O_{1}\right),d\left(O_{2}\right),...,d\left(O_{n}\right)\right)}^{T}.$$

- Step 6: Evaluation of candidates with respect to the objectives.

The objective-based candidates’ evaluation matrix is reformed to the following weighted candidate matrix:

(19) $$W_{candi}=\left[\begin{array}{cccc} W_{candi}{\left(O_{1}\right)}^{T} & W_{candi}{\left(O_{2}\right)}^{T} & \cdots & W_{candi}{\left(O_{n}\right)}^{T} \end{array}\right].$$

- Step 7: Deciding on a solution.

The candidate that has the highest value is selected as the solution by multiplying the two resulting matrices, Equations (18) and (19), composed of weights as follows:

(20) $$Function^{*}=\underset{l}{\mathrm{argmax}}\left(W_{candi}\times W_{obj}^{T}\right).$$

Equation (20) gives the best next point to move to among candidates. FAHP-based decision-making continues until the robot arrives at its destination.

### 4.2. A* Based Path Planning

A* is one of the most famous heuristic path planning algorithms [48]. If the utilized heuristic function is admissible (i.e., it never overestimates the actual cost), A* has been shown to be optimally efficient and is guaranteed to provide an optimal solution. In A*, following terminologies are defined [48]: g(n) is the actual cost of an optimal path from *s* (the starting position) to *n* (any vertex *n*); h(n) is the heuristic estimated cost from *n* to *t* (the target position). The heuristic is normally defined as the distance between current position to the target. To calculate the heuristic, Manhattan distance, diagonal distance, and Euclidean distance are generally used [50]. As it moves from the starting position to the target position, the goal. A* selects the node that has the lowest value in f(n)=g(n)+h(n). The detailed calculation process and implementation are presented in the Algorithm 1.
**Algorithm 1** A* algorithm pseudo code.
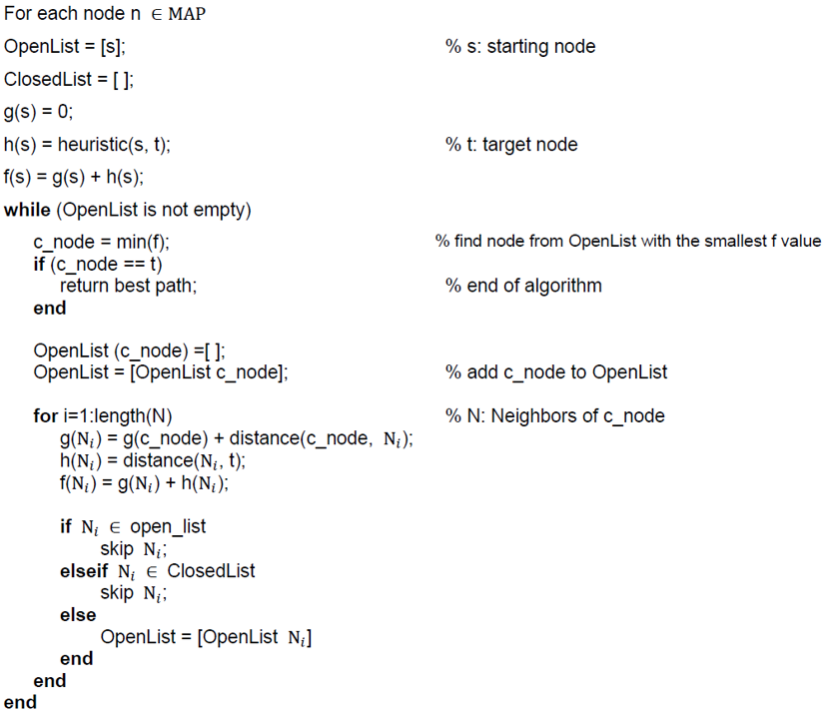


A* ensures the optimal path planning and uses a heuristic (big differences between the Dijkstra’s algorithms) to shorten the path generation time, but the time to calculate the path is still quite long and the execution speed depends heavily on the accuracy of the heuristic function h(n). In addition, A* cannot cope with the varying working spaces and moving obstacles well.

### 4.3. A* and FAHP Hybrid Path Planning

**Figure 6:**
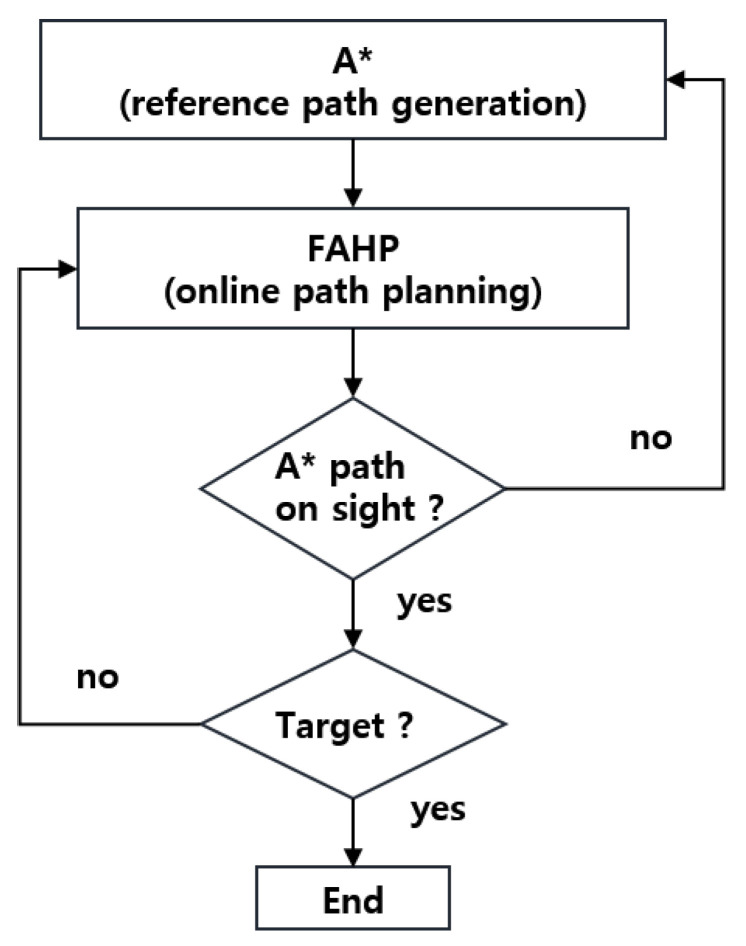
Schematic of the A*–FAHP hybrid path planning algorithm.

A* is widely used because it has excellent performance in searching for an optimal path. However, it has drawbacks, such as taking long time for path generation and lack of avoidance capacity against unplanned obstacles. On the other hand, FAHP selects the optimal solution from the candidates having good path planning performances in the dynamic environment. Therefore, immediate avoidance is possible when dynamic obstacles appear. In some cases, it is possible to plan the optimal path from the origin to the destination through the combination of these local optimal solutions. However, there is no guarantee of optimal path generation from a global perspective. In order to maximize the advantages of A* and FAHP and to compensate for the disadvantages, a hybrid path planning algorithm is proposed, as shown in the Figure 6. At the beginning of the path planning, A* generates an approximate path under scaled-down map to reduce map generation time. Additionally, the FAHP selects a candidate to move considering multiple objects including the closeness to the A*-based path. If the A*-based path does not exist in the candidate set, the A* algorithm plans the path to the target again. Even though the A* path could be inaccurate, FAHP selects an optimal solution among candidates.

**Figure 7:**
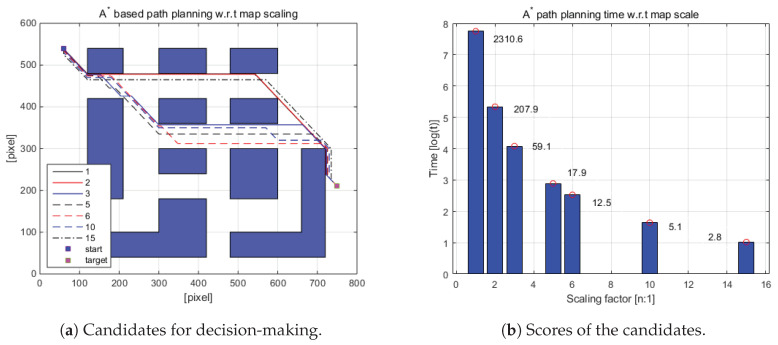
A* path planning time with respect to scaling factors.

In order to reduce the computational time to create a path via A*, the given map is scaled down at the beginning of path planning. Then A* algorithm is applied to the scaled-down map to generate the driving path of the robot [51]. Figure 7a,b shows the scaled-down-map-based navigation performance. In Figure 7a, the blue dot and red dot represent the starting position and the target position, respectively. Firstly, the original map is scaled down to the given ratio, and a path is generated from the reduced map. Then, the generated path is returned to the original map size (800 pixels × 600 pixels), and the driving path generation performance of each scaling factor is compared. As shown in Table 2 and Figure 7b, it is observed that when the original map is reduced by two times, three times, …, 15 times, the path generation time is reduced tremendously as well, i.e., the original map-based path planning takes 2310.6 s, but it takes 2.8 s for 15-times-reduced map. However, the driving path is only 1.22% increased. Considering the reduction in the path calculation time, the distance increase in the path of 1.22% can be regarded as minor. Therefore, a 15-times scaled-down map is used to generate the approximate (or reference) path via A* algorithm.

Once the approximate path is obtained, the local optimal solution is selected by FAHP. In order to implement the FAHP, the following three objectives are considered: closeness to the A* based approximate path $\left(O_{1}\right)$, safety against the obstacle $\left(O_{2}\right)$, and rotation to the target $\left(O_{3}\right)$. The candidates are defined as follows:

(21) $$P_{n}=\theta_{r}-\pi /2+\Delta \theta \times \pi /180\left(n-1\right),$$

(22) $$x_{P_{n}}=x_{r}+r_{s}cos\left(P_{n}\right),$$

(23) $$y_{P_{n}}=y_{r}+r_{s}sin\left(P_{n}\right),$$

where Δθ, $P_{n}$, and ($x_{p_{n}}$, $y_{p_{n}}$) denote the sensor angular resolution, an alternative angle, and an alternative position on $P_{n}$, respectively. $r_{s}$ represents the sensor’s detection range, and the robot’s current position is defined as ($x_{r}$, $y_{r}$,$\theta_{r}$). The first objective, closeness to the A* based approximate path, is given as:

(24) $$O_{1}\left(P_{n}\right)=\sqrt{{\left(opt_{x}-x_{p_{n}}\right)}^{2}+{\left(opt_{y}-y_{p_{n}}\right)}^{2}}.$$

where $\left(opt_{x},opt_{y}\right)$ represents the intersection of the sensing boundary and the approximate path from A*. The second objective, safety against to the target, is defined as:

(25) $$O_{2}\left(P_{n}\right)=\left\{\begin{array}{cc} SM_{min}, & \left(rP_{n}\leq r+r\theta_{min}\right)\\ \frac{1}{r}\left(rP_{n}-r\theta_{min}-r\right)+SM_{min}, & \left(r+r\theta_{min}\leq rP_{n}\leq r\theta_{min}+2r\right)\\ SM_{max}, & \left(r\theta_{min}+2r\leq rP_{n}\leq r\theta_{max}-2r\right)\\ -\frac{1}{r}\left(rP_{n}-r\theta_{min}+r\right)+SM_{min}, & \left(r\theta_{max}-2r\leq rP_{n}\leq r\theta_{max}-r\right)\\ SM_{min}, & \left(rP_{n}\geq r\theta_{max}-r\right) \end{array}\right.$$

where $rP_{n}$ denotes arc length of *n*-th alternative, and r denotes the sensing range of the robot’s distance sensor. The third objective is the rotation angle to the target and is expressed as follows:

(26) $$O_{3}\left(P_{n}\right)=tan^{-1}\left(\frac{y_{t}-y_{p_{n}}}{x_{t}-x_{p_{n}}}\right)-\theta_{r}.$$

After the objectives are defined, the FAHP is implemented following Equations (13)–(20) until the robot reaches the target position. However, as the Figure 6 displays, if there is no intersection between sensing boundary and the A*-based path, the A* algorithm recalculates the path from the current robot position to the target. That is, in case of a sudden obstacle appearance or a planned path departure, a new path from the current robot location to the destination is generated based on the A* algorithm. As described above, it is obvious that a short time is required to generate a new path because the original map is reduced to generate a path to the goal.

## 5. Simulations and Results

In this section, the performances of the suggested control strategy and the path planning algorithm are verified via numerical simulations. The higher-level controller plans the path and the lower-level controller tracks the planned path. In the simulation, the lower-level controller’s performance was verified firstly. Then, the path generation performance of the proposed method was investigated based on the assumption that the path tracking performance had been verified. All simulations were performed on four-wheel OWMR platform kinematics in MATLAB environment. BLS-based control performance was compared to the traditional control methods, PID, in the following scenarios: point-to-point movement, circular path tracking, and random movement target tracking scenarios. The A*–FAHP hybrid algorithm is compared with original A* path planning in three scenarios: a stationary warehouse layout, a dynamic warehouse layout, and an autonomous valet parking scenario. In particular, the valet parking scenario clearly demonstrates the benefit of using OWMR. The dimensions of the valet parking robot are assumed as a circular shape with radius 2.7 m, to cover an area of 2 m × 5 m (width × depth, m). By applying 3% localization uncertainty, the sensor noise and the robot’s internal and external errors were taken into account in the simulations.

### 5.1. Performance of BLS-Based Motion Controller

#### 5.1.1. Point-to-Point Movement

**Figure 8:**
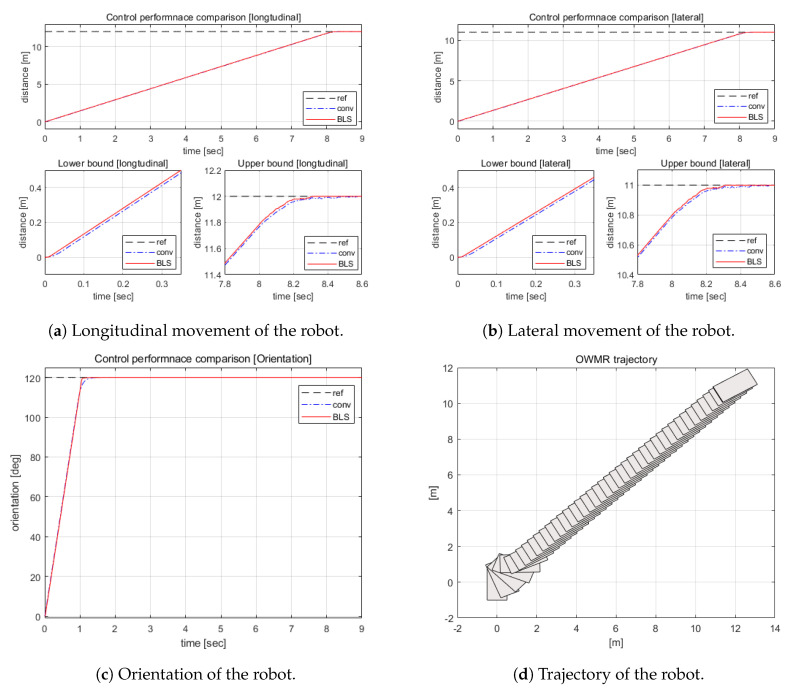
Point-to-point movement of the OWMR.

A position tracking task has been simulated to demonstrate the performance of the suggested control algorithm. The control performance is compared with conventional controller, i.e., the PID controller. The task was to move the robot from the current position, (0,0,0) to target position $\left(12,11,\frac{2}{3}\pi \right)$. As shown in Figure 8a–d, the BLS-based control algorithm outperformed the other in error elimination and settling time. The RMS error of BLS was 5.69, while the PID had an error of 5.70. Additionally, the settling time of BLS was 10.83 (s) while that of conventional controller was 11.02 (s). Under the 3% of position uncertainty, the suggested algorithm successfully tracked the target. Figure 8d shows the characteristics of OWMR: it moved toward the target while keeping the orientation of the robot.

#### 5.1.2. Circular Path Tracking

**Figure 9:**
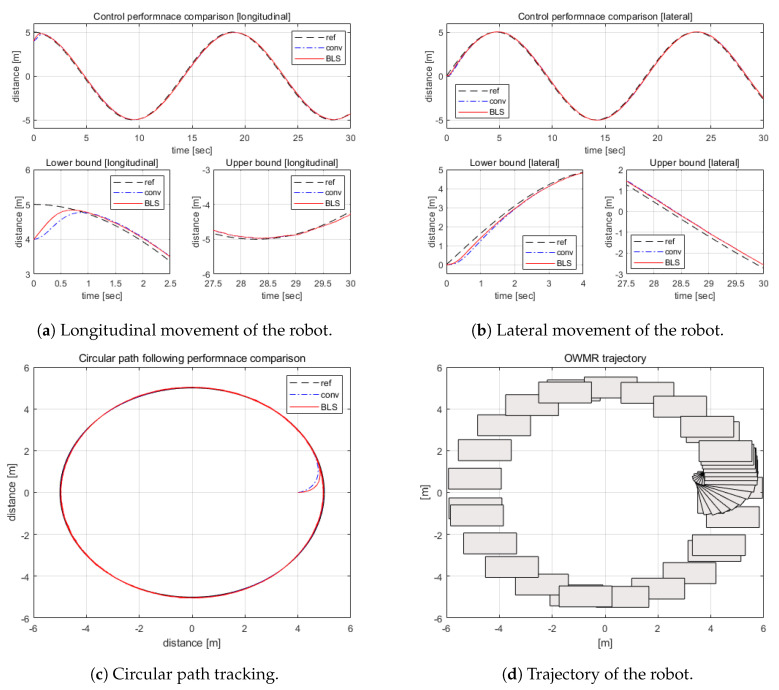
Circular movement of the OWMR.

Next, the circular path tracking control performance was investigated. Figure 9 displays the simulation results. From a–c of Figure 9, it was observed that the proposed motion controller tracked the path faster than the compared control method. In addition, the RMS errors of the BLS control and PID were 0.1886 m and 0.2074 m, respectively, indicating that the error elimination ability of the BLS control is superior to the PID. Additionally, Figure 9d shows the benefit of the OWMR.

#### 5.1.3. Randomly Moving Target Tracking

**Figure 10:**
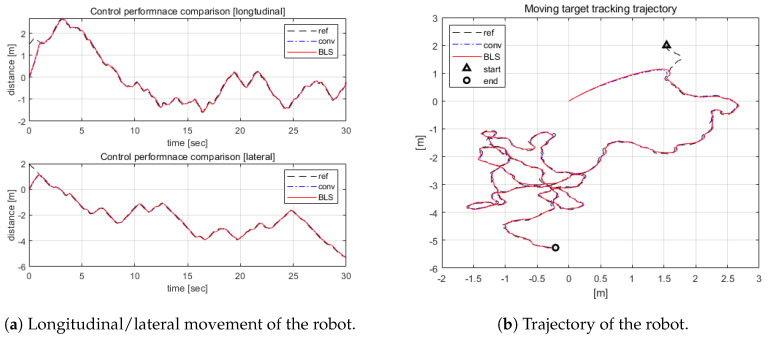
Random movement of the OWMR.

As the most challenging task, randomly moving target tracking is demonstrated herein. The target randomly moved a total distance of 30 m from the starting point to the final position. As shown in Figure 10, it has been demonstrated that BLS-based control has superior performance in terms of target tracking speed and error elimination. When analyzing the simulation results, it was confirmed that the proposed control system and PID showed 0.23 m and 0.33 m RMS error, respectively.

### 5.2. Performance of the Hybrid Path Planning Algorithm

#### 5.2.1. The Hybrid Path Planning Algorithm under the Stationary Working Environment

**Figure 11:**
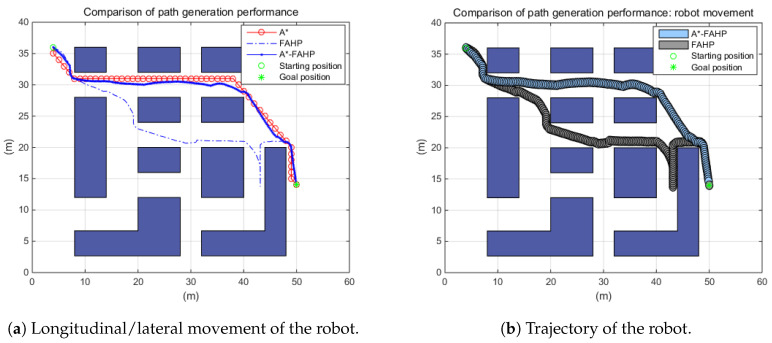
Path planning performance under stationary environment.

The A*–FAHP hybrid path planning algorithm was investigated under the stationary warehouse environment wherein the working space did not change at all. In the unchanging environment, a circular shape OWMR moved from the starting position to the goal position as shown in Figure 11.

Three path planning methods, A*, FAHP, and the A*–FAHP hybrid algorithm, were compared under the same working conditions. As it is known, the A* generates the optimal path to the goal position when the explicit map is given. However, since the FAHP did not have all the information about the working environment, it tracked the goal position via reactive way, and consequently it failed to generate an optimal path from a global point of view. However, the proposed method achieved the shortest travel distance and shortest path generation time by referring to the approximate A* path and selection sub-goals through FAHP. Table 3 shows the path generation time and travel distance of each path planning method. In the case of FAHP, which is an online route planning method, path generation time was not taken into account because the path was planned immediately under the given condition.

#### 5.2.2. The Hybrid Path Planning Algorithm under the Dynamic Working Environment

**Figure 12:**
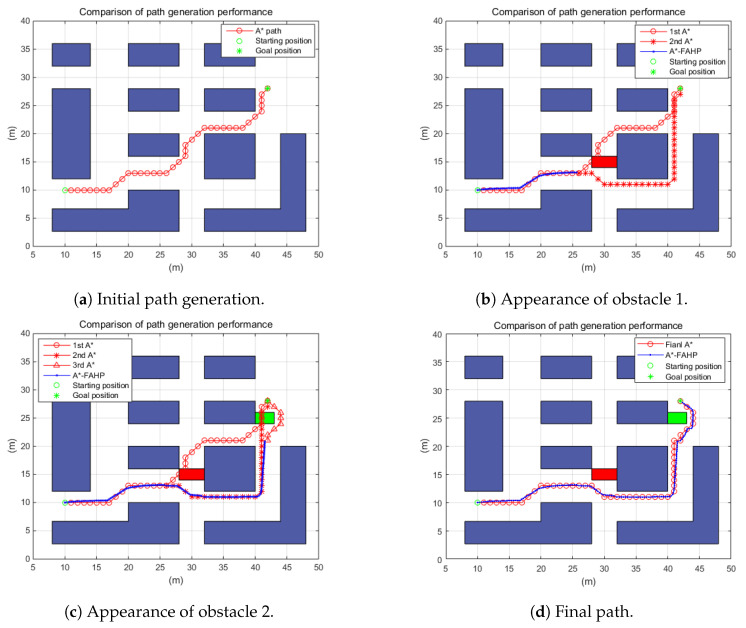
Path planning performance under the dynamic environment.

Under the dynamic warehouse environment, the performance of the suggested navigation algorithm has been tested as well. The warehouse map was the same as in the previous simulations. However, the starting position and the goal position were changed. In addition, as shown in (b,c) of Figure 12, the original path planned without obstacle conditions was blocked by the sudden emergence of an obstacle. In order to avoid collision, the alternative path had to be regenerated according to the A*–FAHP path planning algorithm. Since two obstacles appeared, at least two path regenerations were required. From the simulation, the time consumption for path generation was calculated under a 800 × 600 pixel map. Additionally, it was observed that it took a total of 1947.40 s (1st path generation: 1348.12 (s), 2nd path generation: 577.13 (s), final path generation: 22.15 (s)) to regenerate the path with the original A* algorithm in the given dynamic working environment. However, the suggested algorithm required only 2.19 s (1st path generation: 1.18 (s), 2nd path generation: 0.82 (s), final path generation: 0.19 (s)). Travel distances: that of the final A* was 52.14 m but the travel distance of A*–FAHP was 51.0 m. That was because A* traveled only integer positions while FAHP could move to any position in the map. Through analysis of simulation results in a dynamic warehouse environment, improvements in terms of path generation time and travel distance reduction via the proposed path planning algorithm have been demonstrated.

#### 5.2.3. The Hybrid Path Planning Algorithm under the Autonomous Valet Parking Scenario

**Figure 13:**
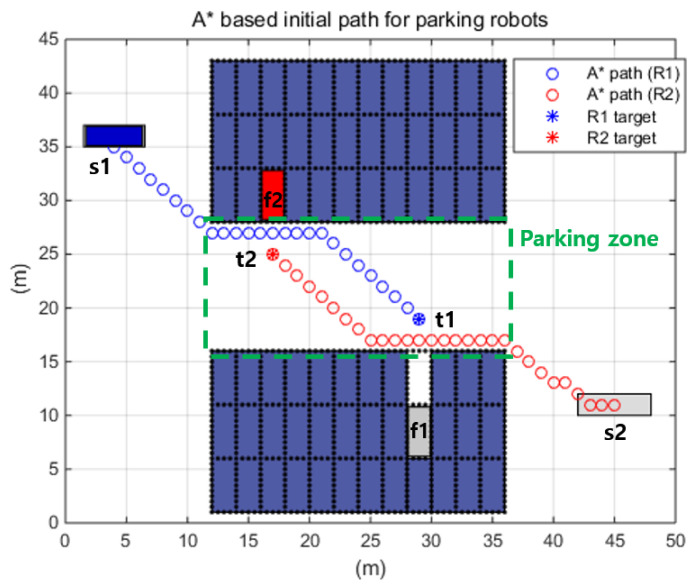
Autonomous parking scenario.

Autonomous valet parking is a potential application of OWRM. Therefore, the final simulation was conducted under an autonomous valet parking scenario. During the simulation, the following things were assumed:

- The autonomous parking management system assigns parking robots (which robot doing what) and notifies the starting/goal position to the assigned robot. Additionally, the path generation is conducted by individual robots via A*–FAHP.
- Individual parking space (black dot boxes in Figure 13) is 2 × 5 m (width × depth) and omnidirectional movement is preferred in parking areas (green dot boxed area in Figure 13) for safety and efficiency reasons.
- The robots communicate each other, sharing the movement information for cooperative decision-making to avoid collision between robots and increase efficiency (more details are given in [36].

In the simulation, a specific mission was assigned to each parking robot, as shown in Figure 13. It was assumed that the R1 robot was given a mission to park the vehicle (v1, located at s1) in the vertical direction at the f1 location, and the R2 robot was assigned an exit mission of the a vehicle (v2, located at f1). Initially, R1 and R2 were in s1 and s2, respectively. The final movements from t1 to f1 and t2 to f2 were not simulated because R1 and R2 reached the vertical areas of t1 and t2. Therefore, in the simulation, the situations wherein R1 moved from s1 to t1 and R2 moves from s2 to t2 were investigated.

**Figure 14:**
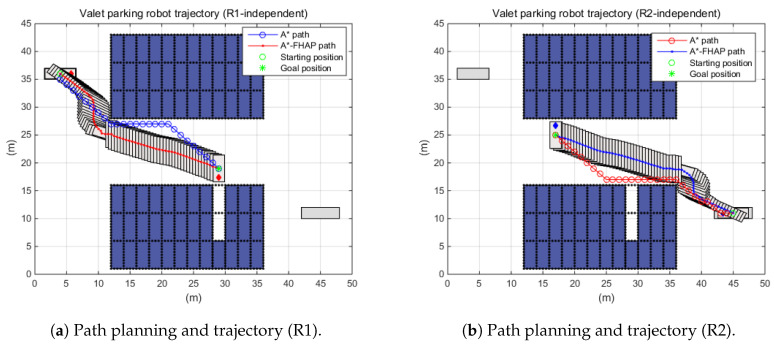
Path planning performance under the autonomous valet parking scenario.

First, the situations in which each robot independently performed a given mission were simulated as shown in Figure 14. In both cases, it was observed that the parking robots successfully moved to the target positions via A*–FAHP. For R1, the travel distance through A* was 32.63 m while that of A*–FAHP was 33.02 m. And R2 traveled 33.80 m through A* but 33.13 m via the suggested algorithm. Although the travel distance was not improved significantly, as shown in the previous simulation results, the path generation time was reduced.

**Figure 15:**
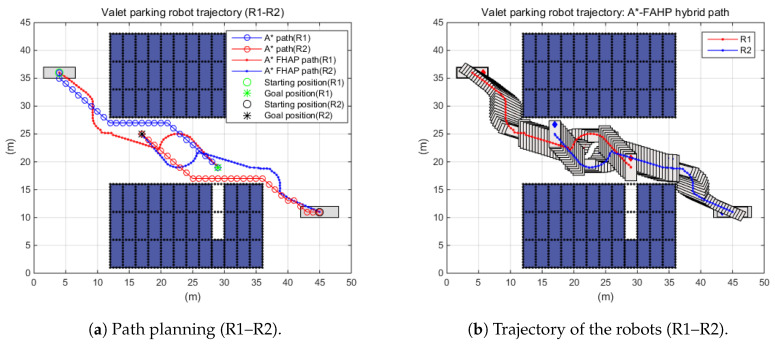
Multi-robot path planning performance under autonomous valet parking scenario.

Indeed, multiple robots worked together to improve the working efficiency in real situations. Figure 15 shows an example of multi-robot interaction. In the simulation scenario, when two robots moved to their destination and recognized each other, the path to avoid collision was regenerated. In the parking area, each robot shared the gains of all movable positions with the opponent robot. Through the sharing of this information, the path generation algorithm determined the optimal movement position (path) to avoid collision between the two robots. The simulation investigated the travel distance for each robot in the R1 and R2 interaction scenarios. By means of the A*–FAHP, two OWMR successfully moved to the target without collision. In addition, the driving characteristics of OWMR helped with improving spacial efficiency in the valet parking scenario. When applying the A* algorithm, R1 and R2 traveled 32.63 m and 33.80 m, respectively, while they moved 36.90 m and 37.54 m through A*–FAHP. The increase in travel distance was caused by path regeneration to avoid collision. The trajectories of each robot are shown in Figure 15.

#### 5.2.4. Analysis of Simulation Results

The performance of the proposed hybrid path planning algorithm was verified under a stationary working environment, a dynamic working environment, and an autonomous valet parking scenario. The biggest advantage of A*–FAHP is that it generates an optimal path in a short-time under a dynamic environment. That is, the optimal path is generated through the A* algorithm, and it is combined with FAHP to achieve the dynamic environment response ability. In order to reduce path generation time, the given map is down-scaled. Through this, the path generation time was significantly reduced while maintaining the path optimality of A*. Therefore, in the analysis of simulation results, the focus was on the time consumption in generating the path rather than the distance traveled. In all simulation cases, the A*–FAHP algorithm took the minimal time compared to other methods for path generation, while short travel distances were observed in several cases. This was because there was no restriction on the direction of movement in FAHP-based path generation, but the A* algorithm only moved in the predefined direction, i.e., the eight directions.

## 6. Conclusions

This research suggests an OWMR control structure that includes the path planning module and the motion control module. A hybrid path generation module is designed to utilize the characteristics of A* and FAHP to generate the optimal path in a short time and secure robustness to a dynamic environment. In addition, the motion control module was developed via brain limbic system control due the advantages of BLS control, such as the fast response and robustness to uncertainty, and error elimination was achieved. In the simulation, the suggested path planning algorithm was compared with A* and the time reduction and optimal path planning performances were verified. In addition, the superiority of the motion controller was demonstrated by comparison to PID control. In all simulations, the suggested algorithm outperformed the conventional control method, PID. From the numerical simulations, it was shown that the proposed control structure successfully controls the OWMR under the dynamic environment. Therefore, the proposed algorithm can be applied not only to the field of logistics to transport materials, but also to various mobile robot applications such as the service robot field that interacts with humans. Due to the proposed algorithm being verified based on simulations, the condition of the road’s surface and failures of the OWMR hardware were not taken into account. Although the proposed algorithm has characteristics suitable for a dynamic working environment, verification and improvement through experiments on problems that may occur in the driving environment of a real robot are essential. The final goal of this study is to develop a practical application of OWMR: an autonomous valet parking robot. To this end, firstly, a small OWMR will be developed to perform verification and improvement of the suggested motion control and path generation algorithm. Additionally, in the future, real system development is planned through joint research with a parking robot system-related company.

## Figures and Tables

**Table 1:** Comparison between online path planning and offline path planning.

	Offline	Online
Method	entire path is generated before the navigation	partial path is generated during the navigation
	- repeatable task and static environment	- dynamic environment
Advantages	- optimal path can be guarantee	- react to the environmental change
		- can work under dynamic environment
		- low computational load
Disadvantages	- high computational time and load	
	- perfect map is required	- locally optimal at best

**Table 2:** Map scaling-based path planning performance comparison.

Scaling Factor	1	2	3	5	6	10	15
time consuming (s)	2310.6	207.9	59.1	17.9	12.5	5.1	2.8
travel distance (pixel)	863.01	864.18	863.6	867.7	868.87	873.55	873.55
distance increase (%)	0.00	0.14	0.07	0.54	0.68	1.22	1.22

**Table 3:** Comparison of path planning performance.

	Time (s)	Travel Distance (m)
A*	2310.6	87.94
FAHP	-	106.36
A*–FAHP hybrid	2.8	87.54

## References

[1] Siegwart R., Nourbakhsh I.R., Scaramuzza D. (2011). Introduction to Autonomous Mobile Robots.

[2] Rojas R., Förster A.G. (2006). Holonomic control of a robot with an omnidirectional drive. KI-Künstliche Intell..

[3] Li X., Zell A. (2009). Motion control of an omnidirectional mobile robot. Informatics in Control, Automation and Robotics.

[4] Liu Y., Zhu J.J., Williams II R.L., Wu J. (2008). Omni-directional mobile robot controller based on trajectory linearization. Robot. Auton. Syst..

[5] Song J.B., Byun K.S. (2004). Design and Control of a Four-Wheeled Omnidirectional Mobile Robot with Steerable Omnidirectional Wheels. J. Robot. Syst..

[6] Hashemi E., Jadidi M.G., Babarsad O.B. Trajectory planning optimization with dynamic modeling of four wheeled omni-directional mobile robots. Proceedings of the 2009 IEEE International Symposium on Computational Intelligence in Robotics and Automation-(CIRA).

[7] Wang C., Liu X., Yang X., Hu F., Jiang A., Yang C. (2018). Trajectory tracking of an omni-directional wheeled mobile robot using a model predictive control strategy. Appl. Sci..

[8] Luo J., Lin Z., Li Y., Yang C. (2019). A teleoperation framework for mobile robots based on shared control. IEEE Robot. Autom. Lett..

[9] Castillo O., Trujillo L., Melin P. (2007). Multiple objective genetic algorithms for path-planning optimization in autonomous mobile robots. Soft Comput..

[10] Masehian E., Sedighizadeh D. A multi-objective PSO-based algorithm for robot path planning. Proceedings of the 2010 IEEE International Conference on Industrial Technology.

[11] Ahmed F., Deb K. (2013). Multi-objective optimal path planning using elitist non-dominated sorting genetic algorithms. Soft Comput..

[12] Ma Y., Hu M., Yan X. (2018). Multi-objective path planning for unmanned surface vehicle with currents effects. ISA Trans..

[13] Yunqiang H., Wende K., Lin C., Xiaokun L. (2018). Research on multi-objective path planning of a robot based on artificial potential field method. Int. J. Wirel. Mob. Comput..

[14] Kim C., Langari R. Analytical Hierarchy Process and Brain Limbic System combined strategy for mobile robot navigation. Proceedings of the 2010 IEEE/ASME International Conference on Advanced Intelligent Mechatronics.

[15] Kouzehgar M., Rajesh Elara M., Ann Philip M., Arunmozhi M., Prabakaran V. (2019). Multi-Criteria Decision Making for Efficient Tiling Path Planning in a Tetris-Inspired Self-Reconfigurable Cleaning Robot. Appl. Sci..

[16] Zagradjanin N., Pamucar D., Jovanovic K. (2019). Cloud-Based Multi-Robot Path Planning in Complex and Crowded Environment with Multi-Criteria Decision Making Using Full Consistency Method. Symmetry.

[17] Kalmár-Nagy T., D’Andrea R., Ganguly P. (2004). Near-optimal dynamic trajectory generation and control of an omnidirectional vehicle. Robot. Auton. Syst..

[18] Choi J.W., Curry R.E., Elkaim G.H. Obstacle avoiding real-time trajectory generation and control of omnidirectional vehicles. Proceedings of the 2009 American Control Conference.

[19] Kong H., Yang C., Li G., Dai S.L. (2020). A sEMG-Based Shared Control System With No-Target Obstacle Avoidance for Omnidirectional Mobile Robots. IEEE Access.

[20] Morén J., Balkenius C. (2000). A computational model of emotional learning in the amygdala. Anim. Animat..

[21] Lucas C., Shahmirzadi D., Sheikholeslami N. (2004). Introducing BELBIC: Brain emotional learning based intelligent controller. Intell. Autom. Soft Comput..

[22] Lucas C., Milasi R.M., Araabi B.N. (2006). Intelligent modeling and control of washing machine using locally linear neuro-fuzzy (llnf) modeling and modified brain emotional learning based intelligent controller (BELBIC). Asian J. Control..

[23] Rouhani H., Jalili M., Araabi B.N., Eppler W., Lucas C. (2007). Brain emotional learning based intelligent controller applied to neurofuzzy model of micro-heat exchanger. Expert Syst. Appl..

[24] Sharbafi M.A., Lucas C., Daneshvar R. (2010). Motion control of omni-directional three-wheel robots by brain-emotional-learning-based intelligent controller. IEEE Trans. Syst. Man, Cybern. Part C Appl. Rev..

[25] Dehkordi B.M., Kiyoumarsi A., Hamedani P., Lucas C. (2011). A comparative study of various intelligent based controllers for speed control of IPMSM drives in the field-weakening region. Expert Syst. Appl..

[26] Kim C., Langari R. (2011). Brain limbic system-based intelligent controller application to lane change manoeuvre. Veh. Syst. Dyn..

[27] Kim C., Langari R. (2012). Adaptive analytic hierarchy process-based decision making to enhance vehicle autonomy. IEEE Trans. Veh. Technol..

[28] Jokar A., Zomorodian R., Ghofrani M.B., Khodaparast P. (2016). Active control of surge in centrifugal compressors using a brain emotional learning-based intelligent controller. Proc. Inst. Mech. Eng. Part C J. Mech. Eng. Sci..

[29] Jafari M., Xu H. (2019). A biologically-inspired distributed fault tolerant flocking control for multi-agent system in presence of uncertain dynamics and unknown disturbance. Eng. Appl. Artif. Intell..

[30] Satty T. (1980). The Analytic Hierarchy Process.

[31] Atthirawong W., MacCarthy B. An application of the analytical hierarchy process to international location decision-making. Proceedings of the 7th Annual Cambridge International Manufacturing Symposium: Restructuring Global Manufacturing.

[32] Locatelli G., Mancini M. (2012). A framework for the selection of the right nuclear power plant. Int. J. Prod. Res..

[33] Chen P.Y., Wu J.K., Pai N.S., Lai Y.C. (2014). Design and Implementation of an Autonomous Parking Controller Using a Fuzzy controller and AHP for Car-Like Mobile Robot. Int. J. Comput. Consum. Control.

[34] Bagga P., Joshi A., Hans R. (2019). QoS based Web Service Selection and Multi-Criteria Decision Making Methods. Int. J. Interact. Multimed. Artif. Intell..

[35] Stanković M., Gladović P., Popović V. (2019). Determining the importance of the criteria of traffic accessibility using fuzzy AHP and rough AHP method. Decis. Mak. Appl. Manag. Eng..

[36] Kim C., Won J.S. (2020). A Fuzzy Analytic Hierarchy Process and Cooperative Game Theory Combined Multiple Mobile Robot Navigation Algorithm. Sensors.

[37] Popovic M., Kuzmanović M., Savić G. (2018). A comparative empirical study of Analytic Hierarchy Process and Conjoint analysis: Literature review. Decis. Mak. Appl. Manag. Eng..

[38] Kim C., Kim Y., Yi H. (2020). Fuzzy Analytic Hierarchy Process-Based Mobile Robot Path Planning. Electronics.

[39] Chan F.T., Kumar N. (2007). Global supplier development considering risk factors using fuzzy extended AHP-based approach. Omega.

[40] Van Laarhoven P.J., Pedrycz W. (1983). A fuzzy extension of Saaty’s priority theory. Fuzzy Sets Syst..

[41] Chang D.Y. (1992). Extent analysis and synthetic decision. Optim. Tech. Appl..

[42] Yoon S.W., Park S.B., Kim J.S. (2015). Kalman filter sensor fusion for Mecanum wheeled automated guided vehicle localization. J. Sens..

[43] Baede T. (2006). Motion control of an omnidirectional mobile robot. Traineesh. Rep. DCT.

[44] Phunopas A., Inoue S. (2018). Motion Improvement of Four-Wheeled Omnidirectional Mobile Robots for Indoor Terrain. J. Robot. Netw. Artif. Life.

[45] Kim C., Langari R. (2013). Application of brain limbic system to adaptive cruise control. Int. J. Veh. Auton. Syst..

[46] Kim C., Langari R. (2011). A mobile robot target tracking via brain limbic system based control. Int. J. Robot. Autom..

[47] Shiller Z. (2015). Off-line and on-line trajectory planning. Motion and Operation Planning of Robotic Systems.

[48] Hart P.E., Nilsson N.J., Raphael B. (1968). A formal basis for the heuristic determination of minimum cost paths. IEEE Trans. Syst. Sci. Cybern..

[49] Chang D.Y. (1996). Applications of the extent analysis method on fuzzy AHP. Eur. J. Oper. Res..

[50] Paden B., Čáp M., Yong S.Z., Yershov D., Frazzoli E. (2016). A survey of motion planning and control techniques for self-driving urban vehicles. IEEE Trans. Intell. Veh..

[51] Ranjitkar H.S., Karki S. (2016). Comparison of A*, Euclidean and Manhattan Distance Using Influence Map in MS. Pac-Man.

